# Investigating the Immunological and Biological Equilibrium of Reservoir Hosts and Pathogenic *Leptospira*: Balancing the Solution to an Acute Problem?

**DOI:** 10.3389/fmicb.2020.02005

**Published:** 2020-08-14

**Authors:** Ellie J. Putz, Jarlath E. Nally

**Affiliations:** Infectious Bacterial Diseases Research Unit, National Animal Disease Center, United States Department of Agriculture, Agricultural Research Service, Ames, IA, United States

**Keywords:** leptospirosis, zoonotic, reservoir host, rat, bovine

## Abstract

Leptospirosis is a devastating zoonotic disease affecting people and animals across the globe. Pathogenic leptospires are excreted in urine of reservoir hosts which directly or indirectly leads to continued disease transmission, via contact with mucous membranes or a breach of the skin barrier of another host. Human fatalities approach 60,000 deaths per annum; though most vertebrates are susceptible to leptospirosis, complex interactions between host species and serovars of *Leptospira* can yield disease phenotypes that vary from asymptomatic shedding in reservoir hosts, to multi-organ failure in incidental hosts. Clinical symptoms of acute leptospirosis reflect the diverse range of pathogenic species and serovars that cause infection, the level of exposure, and the relationship of the pathogen with the given host. However, in all cases, pathogenic *Leptospira* are excreted into the environment via urine from reservoir hosts which are uniformly recognized as asymptomatic carriers. Therefore, the reservoir host serves as the cornerstone of persistent disease transmission. Although bacterin vaccines can be used to abate renal carriage and excretion in domestic animal species, there is an urgent need to advance our understanding of immune-mediated host–pathogen interactions that facilitate persistent asymptomatic carriage. This review summarizes the current understanding of host–pathogen interactions in the reservoir host and prioritizes research to unravel mechanisms that allow for colonization but not destruction of the host. This information is required to understand, and ultimately control, the transmission of pathogenic *Leptospira*.

## Introduction

Global human incidence of acute leptospirosis is estimated at 1.03 million annual cases, with higher incidence in tropical regions and populations from urban slum environments (Costa et al., 2015a; Haake and Levett, 2015). Clinical presentation of leptospirosis can vary from a fever, to widespread vascular damage, to multi-organ failure (De Brito et al., 2018). The highly severe cases of leptospirosis, also known as Weil’s disease, are characterized largely by renal and hepatic injury, but may also include pulmonary and skeletal muscle damage (De Brito et al., 2018). The wide range of leptospirosis symptoms often emulate other diseases, including influenza, dengue fever, and hepatitis, which confounds diagnosis leading to under-reporting of the true incidence of disease (Hartskeerl et al., 2011). More critically, all human cases of leptospirosis are the result of transmission directly (such as exposure from livestock, a pet, wildlife, or rodent pest) or indirectly (contaminated water or soil) from an animal host. In the reservoir animal host, the bacteria colonize the tubular lumen of the kidney, from which they are persistently excreted via urine (Picardeau, 2017). Water and soil that has been contaminated by infected urine represents a major environmental factor that propagates the spread of the bacteria in areas of high prevalence and tropical regions (Ganoza et al., 2006). Livestock exposure and production are considered important risk factors for disease (Mwachui et al., 2015). Positive cases have also been reported after occupational or recreational exposure including veterinary and abattoir work, in rice and banana farmers, as well as after adventure tourism activities such as recreational water sports (Monahan et al., 2009a; Haake and Levett, 2015).

Substantial scientific literature is dedicated to describing human case studies, geographical incidence, and documenting outbreak details. In infected patients, there is wide variability in symptoms and severity of disease (Hartskeerl et al., 2011; Haake and Levett, 2015; Picardeau, 2017), making compilation of human factors into meaningful groups difficult and confounded by uncontrollable human variables (geographical region, weight, race, diet, age, other health factors, etc.). Species, serovar and even strain differences within pathogenic *Leptospira* also complicate the extrapolation of distinct observations. The existence of both genetic and serologic classification schemes can add another level of complexity to interpretation of these observations. Individual *Leptospira* species are represented by multiple serogroups which in turn can include multiple serovars. In addition, certain serovars may also be represented by strains from numerous separate species. For example, serovar Hardjo can include both *Leptospira borgpetersenii* (serovar Hardjo, type Hardjobovis) and *Leptospira interrogans* (serovar Hardjo, subtype Hardjoprajitno), and both *L. borgpetersenii* and *L. interrogans* can belong to the same serogroups including Australis, Hebdomadis, Pyrogenes, and Sejroe (Bharti et al., 2003; Moseley et al., 2018). Broad data has been valuable in identifying risk factors such as rodent infestation and co-grazing of livestock (Costa et al., 2014; Martins and Lilenbaum, 2017), and evaluating the impact and environmental contamination after events like extreme weather or flooding (Togami et al., 2018), but have contributed little to the understanding of the underlying biology of infection. The majority of recent leptospirosis literature describes either clinical human presentation, serological regional demographics, or acute disease modeling in cell culture and hamster models. Human cases are rarely documented unless acute disease symptoms are present which leaves the possibility of asymptomatic carriage largely uninvestigated. Similarly, research that addresses mechanisms of persistent carriage in animals is largely neglected; Table 1 shows a summary of the number of scientific publications compared amongst various search parameters for leptospirosis. Publication numbers were reported after PUBMED^1^ search (see Table 1 for exact search terms). To be included, publications did not require the search term to be in the title and were not excluded based on date or language. Literature concerning leptospirosis and human research (7,002 publications), serology (4,964 publications), and acute disease (1,390 publications) dominated publication numbers while hamster publications numbered 482, and reservoir and maintenance hosts combined yielded only 126 (∼1% of total) publications (see Table 1). The focus of leptospirosis research has consistently been to capture acute disease phenotypes, modeling the disease seen in human case studies (Gomes-Solecki et al., 2017), and leaving behind the classic reservoir host systems.

**TABLE 1:** PUBMED search summary of number of publications per respective Leptospirosis parameter.

**Parameter**	**Specific search terms**	**PUBMED number of publications**	**Percentages of total leptospirosis publications**
Literature describing Leptospirosis, Leptospires, Leptospira, etc.	Leptospir*	12,648 (used in this context as total publications)	
Literature describing reservoir hosts	Leptospir* AND (reservoir host OR maintenance host)	126	126/12648 × 100% = 1.0%
Literature utilizing hamster model	Leptospir* AND hamster	482	482/12648 × 100% = 3.8%
Literature describing human work	Leptospir* AND human	7,002	7002/12648 × 100% = 55.4%
Literature describing acute disease	Leptospir* AND Acute	1,390	1390/12648 × 100% = 11.0%
Literature describing serology, seroprevalence, serotype, etc.	Leptospir* AND Sero*	4,964	4964/12648 × 100% = 39.2%

**Search terms were entered as reported below directly into PUBMED (https://www.ncbi.nlm.nih.gov/pubmed). Papers did not require the term in the title, and were not excluded based on date, language, etc. Searches completed April 29, 2020*.*

The high level of contact between domestic livestock and humans make livestock an important source of exposure for humans. Infection and excretion of leptospires from cattle, swine, sheep, goats, and horses all are well documented (Campos et al., 2017; Pinto et al., 2017). In cattle, leptospirosis can cause abortions, or failure to thrive/stillbirth phenotypes in calves which represents a serious economic cost of disease (Martins and Lilenbaum, 2017). While human health is primary, an important consideration is the animal welfare and economic cost of the disease on the livestock community. In developed countries, large dairies may measure the cost in loss of milk and poor reproductive performance (Guitian et al., 1999), while beef operations lose replacement calves to abortions, both of which cost millions of dollars annually (Bennett and Ijpelaar, 2005). However, in developing countries, the loss of milk producing animals or the failure to breed progeny poses a much more significant burden on the farmer and family those animals were helping to support (Perry and Grace, 2009; Rich and Perry, 2011).

Companion animals can also put their human caretakers at risk, as well as other animals. Dogs can act as maintenance hosts of *Leptospira* and can actively shed pathogens in their urine (Rojas et al., 2010). In a study examining the prevalence of canine leptospirosis in the United States and Canada from 1970 to 1998, veterinary hospitals found the incidence of leptospirosis to be increasing over time and estimated prevalence of cases to be 37/100,000 dogs (Ward et al., 2002). More recent research has indicated that shedding of leptospires in dogs is as high as 8% (Harkin et al., 2003; Rojas et al., 2010). Leptospirosis in cats is rare but seropositivity and kidney disease have been documented (Rodriguez et al., 2014; Talebkhan Garoussi et al., 2015; Ojeda et al., 2018). Even captive reptiles including snakes, lizards, and turtles, test seropositive for exposure to *Leptospira* (Ebani, 2017).

Many species of wild animal can carry and spread pathogenic leptospires to incidental hosts, the most widespread and classic example of which is the rat. The mouse can additionally serve as a *Leptospira* reservoir host, however, research and laboratory work also suggests the mouse model can produce sublethal and acute disease presentations (Gomes-Solecki et al., 2017). The common household infestation and world-wide inhabitance of common rodents contribute substantially to the spread of the disease. Other wild animals with documented culture positive cases include sea lions (Acevedo-Whitehouse et al., 2003), deer (Gay et al., 2014), snakes (Ferris et al., 1961), as well as skunks and opossums (Alexander et al., 1972). Serological evidence of exposure has been documented in feral swine, muskrat (Al Saadi and Podt, 1976), elephants (Oni et al., 2007), raccoons (Allen et al., 2014), and crocodiles (Pérez-Flores et al., 2017).

Research on leptospirosis prioritizes incidence, seroprevalence, vaccine development, and investigating acute presentations of infection, often using the hamster model. In this review, we prioritize research outputs from investigations addressing reservoir hosts of leptospirosis, and emphasize the need for broader utilization of the naturally occurring reservoir hosts in leptospirosis investigations. The term ‘reservoir host’ is used to describe individuals who can asymptomatically maintain, and shed *Leptospira* (Loureiro and Lilenbaum, 2020). For the sake of concision, this review will focus on the cow as an example of an important domestic livestock reservoir host and the rat as the primary and most critical example of a wild animal reservoir host.

## The Complicated Relationship of *Leptospira* and Various Host Species

**FIGURE 1:**
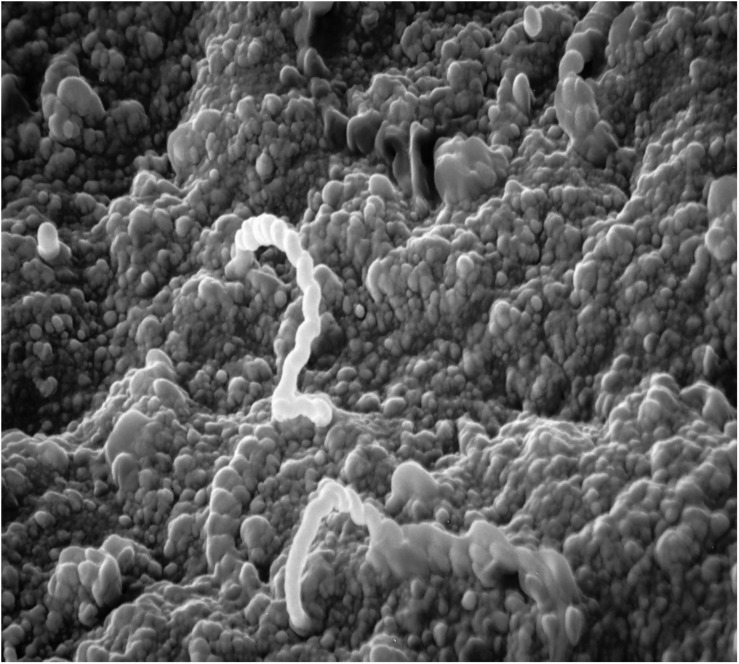
Scanning electron micrograph of adhesion/invasion by *Leptospira interrogans* serovar Pomona (type kennewicki) to equine palpebral conjunctiva. Original magnification 10,000×.

Leptospirosis continues to be one of the most widespread zoonotic diseases, partly because it is strikingly difficult to identify disease, isolate the organism, and prevent by vaccination. Pathogenic *Leptospira* are spread by entry of the pathogen into the bloodstream after either direct or indirect contact with urine or other bodily fluids from an infected host. Exposure of cuts/abrasions with infected urine or soil as well as exposure of any mucous membrane surface, including ocular (Figure 1) and oral routes can result in infection (Faine et al., 1999; Asoh et al., 2014; Sullivan et al., 2017). One of the most unique characteristics of leptospirosis is the host species-specific relationship with different *Leptospira* serovars. Hundreds of serovars are described while whole genome and next-generation sequencing technology have expanded genetic classification to 64 different species of pathogenic and saprophytic *Leptospira* (Kmety and Dikken, 1993; Vincent et al., 2019). Rodents, livestock animals, humans, and companion animals may all respond differently to the same serovar; what may be fatal in canines, may result in severe flu like symptoms in the human, cause an abortion in a cow, and may be completely asymptomatic in the rat. This variability of disease manifestation, dependent on the serovar of *Leptospira* and the host species, may be the most confounding component of leptospirosis. This emphasizes that research findings from one serovar in a particular host should not be extrapolated to the same serovar in other hosts species, or alternative serovars in the same host.

A review of the published literature was used to construct Table 2, which details the conventional disease presentation of common serovars within common host species. Rats were most likely to be designated as an asymptomatic reservoir host, while acute disease was best documented in humans and hamsters. While rats are considered the ‘classic’ reservoir hosts for serovars Icterohaemorrhagiae (Bharti et al., 2003; Gaudie et al., 2008), and Copenhageni (Gomes-Solecki et al., 2017), it is cattle that are considered classic reservoirs of serovar Hardjo (Rajala et al., 2017; Loureiro and Lilenbaum, 2020), pigs (Srinivas et al., 2013; Pedersen et al., 2017) and horses (Ellis et al., 1983) of serovar Bratislava, and dogs of serovar Canicola (Lau et al., 2017; Miotto et al., 2018). *L. interrogans* serovar Lai is highly virulent in human patients, yet is frequently asymptomatic in mice (Li et al., 2010). When cultured with macrophages, serovar Lai was sequestered by vacuoles in murine-derived cells in contrast to human-derived cells where it was maintained free in the cytosol. This difference was correlated with greater pathogen survival in human-derived macrophages, while serovar Lai did not survive in murine-derived macrophages and co-localized more frequently with lysosomal markers (Li et al., 2010). At the hematology level, leptospirosis can cause hemoglobinemia and hemolysis and/or damage of red blood cells (RBCs). Interestingly, this phenomenon is also host species specific. Calves challenged with *L. interrogans* serovar Pomona resulted in hemoglobinemia and RBC damage and abnormalities (Thompson, 1986), while these features are not reported in the hamster, despite the fact that hamsters are still susceptible to Pomona (Srinivas et al., 2013; Techawiwattanaboon et al., 2019). In contrast, infection with *L. interrogans* serovar Ballum results in the opposite, with hemoglobinemia and morphological changes seen in the RBCs of the hamster and not in cattle (Thompson and Manktelow, 1986). Thus, host-species variation in disease presentation may partially stem from recognition and response to the pathogen.

**TABLE 2:** Matrix of relevant host species and *Leptospira* serovar interactions.

***Leptospira* Serovar**	**Host species**						
	**Human**	**Rat**	**Cow**	**Horse**	**Pig**	**Dog**	**Hamster**
Pomona	A (Natarajaseenivasan et al., 2005; Ramirez-Ramirez et al., 2015)	SP (Boey et al., 2019)	SP (Campos et al., 2017) A (Ojeda et al., 2018; Loureiro and Lilenbaum, 2020)	SP (Hamond et al., 2015; Simbizi et al., 2016) A (Hamond et al., 2015)	R (Gay et al., 2014) SP (Lee et al., 2017) A (Mitchell et al., 1966)	A (Adin and Cowgill, 2000; Greenlee et al., 2005) SP (Goldstein et al., 2006)	A (Srinivas et al., 2013; Techawiwattanaboon et al., 2019)
Tarassovi	A (Maze et al., 2016)	SP (Boey et al., 2019)	SP (Campos et al., 2017)	SP (Simbizi et al., 2016)	SP (Lee et al., 2017) R	SP (Barmettler et al., 2011)	NE
Hardjo	A (Bolin and Koellner, 1988; Ezeh et al., 1991) R	SP (Boey et al., 2019)	R* (Ellis et al., 1981; Ellis et al., 1986; Rajala et al., 2017; Loureiro and Lilenbaum, 2020) SP (Campos et al., 2017; Nally et al., 2018a)	A (Hamond et al., 2015) SP (Simbizi et al., 2016)	SP (Hathaway et al., 1983; Lee et al., 2017)	SP (Goldstein et al., 2006; Harland et al., 2013)	A (Zuerner et al., 2012; Srinivas et al., 2013) R (Zuerner et al., 2012; Nally et al., 2018a)
Grippoty phosa	A (Ramirez-Ramirez et al., 2015)	SP (Felt et al., 2011; Boey et al., 2019)	SP (Campos et al., 2017) A (Chiebao et al., 2015; Loureiro and Lilenbaum, 2020)	SP (Simbizi et al., 2016)	SP (Lee et al., 2017)	A (Greenlee et al., 2004) SP (Goldstein et al., 2006)	A (Srinivas et al., 2013)
Icterohae morrhagiae	A (Gaudie et al., 2008; Ramirez-Ramirez et al., 2015)	R* (Gaudie et al., 2008; Lau et al., 2017) SP (Boey et al., 2019)	SP (Campos et al., 2017) A (Ellis and Michno, 1976)	SP (Simbizi et al., 2016)	SP (Lee et al., 2017)	A (Arimitsu et al., 1989) SP (Barmettler et al., 2011)	A (Arimitsu et al., 1989; Srinivas et al., 2013)
Ballum	A (Ferris et al., 1961)	R* (Hathaway and Blackmore, 1981; Schuller et al., 2015) SP (Boey et al., 2019)	SP (Yupiana et al., 2019)	SP (Jung et al., 2010)	SP (Yupiana et al., 2019)	SP (Schuller et al., 2015)	A (Matsui et al., 2015)
Wolffii	A (Zakeri et al., 2010) SP (Zakeri et al., 2010)	SP (Boey et al., 2019)	SP (Campos et al., 2017)	SP (Siqueira et al., 2019)	NE	SP (Zakeri et al., 2010)	SP (Tabata et al., 2002)
Canicola	A (Natarajaseenivasan et al., 2005; Ramirez-Ramirez et al., 2015)	SP (Boey et al., 2019) R (Shenberg et al., 1977)	SP (Zacarias et al., 2008; Campos et al., 2017) R (Zacarias et al., 2008)	SP (Jung et al., 2010; Simbizi et al., 2016)	SP (Lee et al., 2017)	A (Arimitsu et al., 1989) R* (Lau et al., 2017; Miotto et al., 2018) SP (Goldstein et al., 2006)	A (Arimitsu et al., 1989; Srinivas et al., 2013)
Bratislava	SP (Cacciapuoti et al., 1982)	SP (Boey et al., 2019)	SP (Campos et al., 2017)	A (Hamond et al., 2015) R* (Ellis et al., 1983) SP (Jung et al., 2010; Simbizi et al., 2016)	R* (Srinivas et al., 2013)	A (Adin and Cowgill, 2000) ND (Greenlee et al., 2005)	SP (Sebek et al., 1987)
Copen hageni	A (Zacarias et al., 2008)	R* (Gomes-Solecki et al., 2017) SP (Boey et al., 2019)	R (Zacarias et al., 2008)	SP (Jung et al., 2010)	SP (Kazami et al., 2002)	A (Larson et al., 2017) SP (Harland et al., 2013)	A (Evangelista et al., 2017)
Lai	A (Natarajaseenivasan et al., 2005; Li et al., 2010)	SP (Boey et al., 2019)	NE	SP (Jung et al., 2010)	NE	NE	A (Lambert et al., 2012)
Sejroe	A (Natarajaseenivasan et al., 2005; Maze et al., 2016)	NE	SP (Khamassi Khbou et al., 2017; Vidal et al., 2017)	SP (Jung et al., 2010)	NE	R (Scanziani et al., 1995)	A (Barbosa et al., 2019)

Relative disease presentation of common serovars in various host species is described. Serovar generalizations include literature across type, strain, and geographical region. A, acute disease (includes all acute disease symptoms such as inflammatory response, fevers, lesions, includes severe cases and renal failure); R, reservoir host (bacterial colonization is evident, but otherwise asymptomatic); SP, seropositive (serology identifies positive antibodies against given serovar but clinical details are unknown); ND, no disease (no disease results after exposure or challenge); NE, no evidence (evidence not found in the literature by PUBMED search). The ‘classic’ reservoir host for the given serovar is designated with R*.

It is hypothesized that the result of acute or chronic leptospirosis infection may be partially determined by how promptly and severely the immune system can respond to, and control invading *Leptospira*. Immune cells recognize invading pathogens through pathogen-associated molecular patterns (PAMPs), such as lipopolysaccharide (LPS) from *Leptospira*, with Nod-like or Toll-like receptors (NLRs or TLRs, respectively). TLR4 is regarded as the classic LPS receptor, however, leptospiral LPS is substantially less reactive than comparative Gram-negative bacterial LPS such as *E. coli*. TLRs are expressed on renal epithelial cells and can be activated by leptospiral outer membrane lipoproteins including LipL32 (Monahan et al., 2009b). The role of TLRs in immune response to *Leptospira* has recently received more attention utilizing the tools and the availability of diverse genetic backgrounds in the mouse model (Gomes-Solecki et al., 2017). While murine cells utilized both TLR2 and TLR4 to respond to *Leptospira* LPS, they required TLR4 to respond to LPS membrane component lipid A (Nahori et al., 2005). Others have found that in response to *L. interrogans* serovar Autumnalis (strain 56606) mice utilize only TLR4 (Xia et al., 2017). Curiously, human cells largely failed to be stimulated by *Leptospira* lipid A, and rather have been shown to primarily utilize TLR2 activity against leptospiral LPS (Nahori et al., 2005). In addition to human and mice, porcine and bovine cells also respond to *Leptospira* LPS through TLR2 (Guo et al., 2015, 2016; Vernel-Pauillac and Werts, 2018). In a study comparing the TLR2 profiles of mice (resistant) and hamsters (susceptible) against *L. interrogans* serovar Autumnalis, mice showed a quick induction of TLR2 while hamsters had a significantly delayed TLR2 response in the kidney, liver, and the lung (Zhang et al., 2016). Interestingly, when the hamsters were coinfected with TLR2 agonist Pam3CSK4 and challenge, hamster survival was increased and kidney, liver, and lung lesions were lessened suggesting that TLR2, and the timing of its response, has a role to play in the control of leptospirosis (Zhang et al., 2016).

The role of TLRs and leptospiral detection has interesting implications across different host species. Many TLRs are known to have species specific structure, response, and molecular dependency (Keestra et al., 2008; Werling et al., 2009; Liu et al., 2010; Willcocks et al., 2013). Human and bovine TLR2 for instance, while similar in their Toll interleukin-1 receptor (TIR) domain, have very different extracellular domains, which results in structural modifications that can affect ligand binding and will vary their strength of activation responses to microbial stimuli (Willcocks et al., 2013). The classic LPS receptor TLR4 also exhibits species specific behavior between humans and cattle. While peak TLR4 activation is achieved most often with an LPS-receptor complex including both molecules CD14 and MD2, MD2 was more critical to NF-κB activation in bovine systems than in human ones, while human CD14 had a more prominent role than in cattle (Lizundia et al., 2008). Bovine and humans only share 77 and 76% amino acid homology, and bovine and murine only share 68 and 66% homology for TLR2 and TLR4, respectively (Werling et al., 2006). Even within species, genetic differences in TLR composition may impact immunological traits. TLR2 polymorphisms were identified within dairy cow breeds that were found to have associations with somatic cell scores, which are classic indicators of bovine mastitis most often caused by bacterial infections (Zhang et al., 2009). With the addition of tools such as a new bovine reference genome (Rosen et al., 2020), and the adoption of cattle as a model reservoir host, we are well poised to further investigate these interactions. These unique immunological features stress that within-host observations should not be extrapolated to other species. For instance, the *Leptospira* LPS and TLR2 interactions described above in hamsters, mice, and humans may not apply to bovine TLRs. Even within the bovine experimental model, serovar Hardjo LPS may be recognized differently than that of serovar Autumnalis. The initial detection of *Leptospira* during infection represents a critical juncture for the severity and progression of disease. By comparing the mechanisms of detection between reservoir and acute host species, we can elucidate the methods of immune escape in the reservoir host and may be in a better position to develop preventative and treatment therapeutics targeted toward pathogen detection and clearance. Clearance of the pathogen in reservoir hosts is the ultimate goal to effectively control disease transmission.

Exploring the intricacies behind host immunology and *Leptospira* species-specific interactions justifies the attention of the research community. Clearly a unique relationship between serovars and specific host species exist. However, some strains, within the same serovar, also associate differently between hosts species (Arent et al., 2016, 2017). In a study utilizing restriction endonuclease analysis (REA), ten separate restriction enzyme patterns were found between the two closely related serovars Bratislava (three strains) and Muenchen (seven strains) (Arent et al., 2016). From the isolates analyzed, these patterns were found to be associated geographically, and within specific host species. From the Bratislava serovar strains, one REA pattern was found associated with dogs and horse isolates while another was found exclusively in clinical pig samples (Arent et al., 2016). Of the Muenchen serovar strains, one REA pattern was found associated with pigs and small rodents while most of the others were only found in wildlife samples (Arent et al., 2016). Similarly, work detailing *L. interrogans* serovar Pomona (type Kennewicki) showed distinct restriction fragment length patterns associated with the domestic host species from which isolates were collected (Bolin and Zuerner, 1996). Interestingly, the wildlife samples collected shared restriction patterns, but were associated with the same domestic animal restriction pattern profile at the location (specific farm or herd) from which they were captured (Bolin and Zuerner, 1996). This suggests that strain and host species specificity exist and must be accounted for in scientific observation. This type of epidemiologic analysis has crucial implications for the identification of strain specific host reservoirs, and the classification of strain specific risk for domestic livestock versus wildlife. Experimental work has also confirmed within serovar, strains of *Leptospira* can produce different presentations of disease. Species *L. borgpetersenii*, strains HB203 and JB197, both serovar Hardjo, have nearly identical gene content, and yet HB203 colonizes the kidney of the hamster and produces a chronic infection, while JB197 produces a severe acute infection (Zuerner et al., 2012). Even within strain, comparing a challenge of a virulent versus passage-attenuated strain of Pomona (AKRFB) in bovine macrophages revealed that the challenge with the virulent strain resulted in increased gene expression of IL-10 and higher pathogen internalization by the macrophages compared to the attenuated strain (Nagel et al., 2019).

Broad serovar testing fails to capture such subtle nuances described above, and as such can propagate inaccurate species-specific information (such as expected disease presentation). While emphasis is often placed on which serovars are important for inclusion in a vaccine for a designated animal species, the strain of *Leptospira* included in the vaccine may be equally critical. Although pangenome studies continue to expand our knowledge of *Leptospira* species, there is a need for ‘pan-serovar’ studies to illustrate and characterize within serovar, strain-specific and host species interactions. In fact, as REA data suggests, it may be prudent within leptospiral genomics to analyze whole closed genomes versus contig constructs in an effort to better identify strain nuances. Indeed, the key to vaccine efficiency and cross-protection may have to be resolved at a more strain specific level, which can be elucidated with the aid of appropriate reservoir host models.

## Model Reservoir Hosts: the Cow and the Rat

The most defining feature of a reservoir host is that when infected, they actively shed bacteria while remaining asymptomatic. The most defining feature of *Leptospira* in a reservoir host, is successful colonization of the kidney while escaping a fully fledged immune response that could result in respective host pathology or pathogen clearance. Even upon initial infection, true reservoir hosts lack the fever, cytokine storms, innate immune signals, and other hallmarks of leptospirosis observed in incidental hosts. Antibody production may still be induced in reservoir hosts, but not necessarily. In cattle, there is ample evidence of culture positive animals that were seronegative by MAT (Miller et al., 1991; Nally et al., 2018a), and there are also examples of infected turtles that are PCR positive/MAT (microscopic agglutination test) negative (Oliveira et al., 2016). Bovine neutrophils co-cultured with *L. borgpetersenii* (serovar Hardjo) also minimally produce Neutrophil Extracellular Traps (NET) compared to *Escherichia coli* and PMA controls (Wilson-Welder et al., 2016). In rats, gene expression of immune related genes in spleens of *L. interrogans* (serovar Copenhageni) challenged animals were nearly identical to the spleens on non-infected controls (Nally et al., 2018b). These observations exemplify the delicate immunological and biological equilibrium of persistent leptospirosis in reservoir hosts. Our limited understanding of this relationship represents a significant challenge for the leptospirosis research community.

Cattle represent a common livestock reservoir host with close human interaction. A recent survey of beef cattle found 7% were urine leptospire positive for serovar Hardjo (Nally et al., 2018a, 2020), and small scale dairies have been shown to harbor up to 13–36% serovar Hardjo seropositive cow populations (Rajala et al., 2017; Yupiana et al., 2019) which leaves farm workers and healthy animals vulnerable to exposure. Instead of the multi-organ failure seen in other species with leptospirosis, infected bovine symptoms are predominantly reproductive. Bovine leptospirosis can result in embryonic death, premature birth, altered estrus cycles, abortions, or calves with a failure to thrive (Martins and Lilenbaum, 2017; Loureiro and Lilenbaum, 2020). Since these symptoms are difficult to identify and quantify, detection of disease continues to be a problem for beef and dairy farmers. This also makes cattle difficult to classify as reservoir hosts versus simple incidental hosts. Cattle are regarded as true reservoir hosts for serogroup Sejroe, but may act as incidental hosts for Pomona, Icterohaemorrhagiae, and Grippotyphosa serovars (Martins and Lilenbaum, 2017; Loureiro and Lilenbaum, 2020) (see Table 2). At large, a reservoir host is associated with bacterial colonization of the kidney and subsequent shedding in the urine but research has also demonstrated that the reproductive and genital tracts can carry and harbor the pathogen, as recently reviewed (Loureiro and Lilenbaum, 2020). This has been most articulated in cattle, but also documented in sheep and horses (Arent et al., 2013; Hamond et al., 2015). While classic modes of infection can still result in genital tract colonization, asymptomatically infected breeding animals can also transmit the disease to one another through semen (live cover and artificial insemination, male to female) and vaginal discharge (female to male) (Loureiro and Lilenbaum, 2020). Since bovine disease presentation is most often reproductive in nature, the interaction of *Leptospira* and the genital and reproductive tract is a critical component of study.

Rats were identified as reservoir hosts for leptospirosis as early as 1917, where it was established that rats were important asymptomatic carriers of the disease, and urine or kidney lysate from infected rats was fatal to guinea pig hosts (Ido et al., 1917). While adult rats are expected to be asymptomatic when challenged with most *Leptospira*, suckling rats under 2 weeks of age can show severe signs of disease including jaundice, weight loss, and mortality (Muslich et al., 2015). These symptoms, however, though they appear to be age and dose dependent, were largely not seen after weaning at 23 days of age (Muslich et al., 2015). While there does not appear to be an association with the sex of the rat and bacterial load/infection across numerous species, there is evidence to support a correlation of infection with age of the rat, varying within low, medium, and high incidence (Smith et al., 1961). Different species of rat such as the black rat (*Rattus rattus*) and the brown rat (*Rattus norvegicus*) may inhabit slightly different ecological niches; the black rat can be found in rural farm settings and woodlands while the brown rat is more often identified in close proximity to high population human areas (Hathaway and Blackmore, 1981). As a result, seroprevalence may vary between rat species, even within the same geographical regions (Hathaway and Blackmore, 1981). Rat infestation is considered a major factor associated with leptospirosis (Costa et al., 2014). Rat populations are healthy across the globe and regional surveys have found rats to be seropositive to most common serovars of *Leptospira* (Table 2) (Boey et al., 2019). Seroprevalence has even been reported as high as 100% in countries such as Brazil, and is estimated between 44 and 65% in the United States (Boey et al., 2019). Rats are considered true reservoir hosts of serovar Icterohaemorrhagiae (as well as Copenhageni); experimentally infected rats shed serovar Icterohaemorrhagiae for 220 days (the duration of the experiment), compared to serovar Grippotyphosa which was only shed for approximately 40 days post challenge and then seemingly cleared (Thiermann, 1981). High correlations exist between the *Leptospira* load in the kidney of the rat and the amount of leptospires shed in the urine (Costa et al., 2015b). For the Norway rat (*R. norvegicus*), positive kidneys have average concentrations of 5.9 × 10^6^ leptospires per mL compared to the averaged matched urine samples of 6.1 × 10^6^ leptospires per mL (Costa et al., 2015b) with shedding reports as high as 10^7^ leptospires per mL (Monahan et al., 2008; Costa et al., 2015b; Gomes-Solecki et al., 2017). Rats are social creatures, and individual contact within rat groups can quickly facilitate the spread of disease within a colony. The nature of rats as pests make them difficult to keep out of places like households and barns, and their ability to stow away aboard travel vessels like trucks and boats make rats prime spreading agents of leptospirosis. Though wild rats are primarily responsible for the spread of leptospirosis, pet and laboratory rats are also reservoirs and have been linked to human infection (Gaudie et al., 2008; Boey et al., 2019). Despite the unique relationship of leptospires with rat hosts, most leptospirosis research is conducted with susceptible hamster models that better demonstrate acute parameters of clinical disease. Our assertion is that a better understanding of the interaction of *Leptospira* within the rat host is needed for more rational disease control efforts.

In comparison to the hamster, the rat has more extensive genetic reference tools (Grassmann et al., 2017; Shimoyama et al., 2017), more immunological kits and reagents available, and substantially more genetic models to utilize. Outbred rats are routinely used to model persistent renal carriage (Tucunduva de Faria et al., 2007; Athanazio et al., 2008; RamachandraRao et al., 2015). Inbred rats (*R. norvegicus*) offer additional insights (Nally et al., 2018b); upon challenge with *L. interrogans* (serovar Copenhageni) inbred rats present asymptomatically, but kidneys become colonized and leptospires are shed in urine by 3 weeks, through at least 8 weeks, post challenge. Inbred rats developed robust antibody responses to leptospiral antigens, positive MAT titers, and showed prominent B cell proliferation when stimulated with leptospiral outer membrane proteins (Nally et al., 2018b). Interestingly, despite pathogen presence, the rats were largely void of lesion pathology in kidney tissue (including a lack of interstitial nephritis), and gene expression analysis of prominent T cell pathways between the spleens of infected and non-infected controls yielded few differences (Nally et al., 2018b). This suggests complex immune escape of the pathogen including limited immune activation from the host, however, activated lymphocytes including CD4+ were significantly expanded in the renal lymph nodes of infected animals compared to controls (Nally et al., 2018b). This offers a prime example of the intricacies of the systemic and localized interaction of the pathogen with the reservoir host. The relationship of draining lymph nodes and infected organs could also be explored in the bovine model to determine if this is a characteristic of reservoir hosts, or a unique feature of resistant rodents. If immune cell activation and expansion is not achieved in infected organs, by what mechanisms are the *Leptospira* escaping surveillance and if detection could be achieved, could the pathogen be cleared? The biological and immune signaling that must occur to create the asymptomatic disease presentation is unique to reservoir hosts, highly species specific, and needs to be addressed with appropriate reservoir host models.

While vaccines for leptospirosis in domestic animals exist, they are comprised of whole-cell killed bacterins that are regionally developed for specificity, and suffer from a lack of cross protection between serogroups (Bashiru and Bahaman, 2018), and limited cross protection between serovars (Bouvet et al., 2019). This has especially been illustrated in the creation of a Hardjo cattle vaccine whose efficacy is largely influenced by the type and strain of serovar Hardjo used in the vaccine construction, versus the type and strain of the serovar Hardjo used for experimental challenge. This offers further evidence for the need to expand our current understanding of the relationship between host species, and *Leptospira* species, serovar, and strain. For instance, pentavalent commercial cattle vaccines have been designed to protect against numerous serovars including Canicola, Pomona, Icterohaemorrhagiae, Hardjo (*L. interrogans*), and Grippotyphosa (*Leptospira kirschneri*). However, this pentavalent combination was not protective for cows against challenge with serovar Hardjo (*L. borgpetersenii*), and still resulted in abortion, urinary shedding, and even calves born with *Leptospira* colonized kidneys (Bolin et al., 1989a). Including serovar Hardjo (*L. borgpetersenii*) within a pentavalent vaccine also did not confer protection against the same challenge, despite higher agglutination titers in animals that received the Hardjo-Bovis type vaccine compared to those receiving a Hardjo-Prajitno type vaccine (Bolin et al., 1989b). A monovalent vaccine study that addressed two concentrations of the amount of Hardjo (*L. interrogans*) organisms included in the vaccine formation also failed to confer protection when challenged with Hardjo (*L. borgpetersenii*) (Bolin et al., 1991). However, a monovalent commercially available Hardjo (*L. borgpetersenii*) vaccine was shown to successfully protect cattle against kidney colonization and prevented urinary shedding from a Hardjo (*L. borgpetersenii*) challenge (Bolin and Alt, 2001). This further illustrates the highly specific and complex relationship of the pathogen with host immune recognition. Concerns over a lack of cross protection between serovars, and short lived immunity, have driven attempts utilizing alternative vaccine developments such as recombinant and DNA vaccines (Mwachui et al., 2015; Silveira et al., 2017; Bashiru and Bahaman, 2018). Indeed, live attenuated vaccines have been used successfully to lower the abortion rate in a leptospirosis positive cattle herd (Kenzy et al., 1961) and to protect swine against experimental disease challenge (Fish and Kingscote, 1973), however, the inconsistency in preparation and pathogenicity have left live vaccines mostly unutilized.

The serovar sensitivity, lack of cross protection, and highly specific antibody responses of vaccine challenges imply that a humoral response is a critical driver for complete and long-term host protection. For instance, IgA antibody supplementation has been shown to sufficiently protect guinea pigs from lethal challenge (Jost et al., 1986). In cattle however, cell mediated immune responses also play a role in a successful vaccine response against leptospirosis. Both humoral and cell mediated responses have T cell driven components; Th2 (humoral) and Th1 (cell-mediated) responses, respectively, which are each associated with different cytokine and antibody production. Cattle vaccinated with protective monovalent vaccine against Hardjo type Bovis, produced both IgG1 (Th2) and IgG2 (Th1) antibodies (Brown et al., 2003), suggesting both Th1 and Th2 responses at work. However, protected cattle were also shown to have sustained peripheral blood mononuclear cell proliferation and increased IFN-γ cytokine production, a hallmark of a Th1 cell mediated response, from CD4 and γδ+ T cells (Naiman et al., 2001; Brown et al., 2003), while production of IL-4, a hallmark of Th2 response, was not produced (Brown et al., 2003). Interestingly, cells from the Hardjo monovalent vaccinated cattle also showed a proliferation and IFN-γ response after *in vitro* exposure to *L. kirschneri* serovar Grippotyphosa, suggesting potential Th1 cross protection (Brown et al., 2003). While not a T cell, Natural Killer (NK) cells are also immune cell producers of IFN-γ and have been shown to produce IFN-γ in a ‘memory’ recall-like fashion in response to Hardjo monovalent leptospirosis vaccination and challenge (Zuerner et al., 2011). This work suggests an important role for IFN-γ and cell mediated immunity in the context of a protective vaccine response, however, apprehension remains about how long-lived this protection is. Collectively, this highlights a gap in current research that both humoral and cell mediated immunology responses should be included in the design of prevention and treatment technologies. The mechanisms which drive Th1 and Th2 responses, critical cytokine production, and different immune cell differentiation and activation requires better characterization. These effects are likely species specific, and possibly additionally influenced by the age of the host. For instance, in neonatal cattle and other ruminants, γδ T cells dominate the T cell profile, contributing as high as 60% of total peripheral blood mononuclear cells, however, as the animals develop, the proportion of γδ T cells may constitute 10–40% of peripheral blood cells (Hein and Mackay, 1991; Holderness et al., 2013; Guerra-Maupome et al., 2019). In contrast, γδ T cells in humans and mice constitute 0.5–10% of peripheral blood cells, and in rats only 1–5% (Holderness et al., 2013). γδ T cells are producers of IFN-γ, and capable of producing memory γδ T cell populations in response to *Leptospira* vaccination (Naiman et al., 2002; Blumerman et al., 2007), indicating a potential vaccine target, especially considering the bovine model which have such high γδ T cell numbers in circulation. Similar to cattle, pigs have elevated percentages of γδ T cells (30% of peripheral cells), but also maintain a population of circulating double positive CD4 + CD8+ T cells (not present in humans and mice) whose percentages increase with the age of the pig (Cheon et al., 2014). These double positives share effector and memory phenotypes and can be found in both blood and colostrum (Hlavova et al., 2014), which might have different vaccine implications for swine. Such examples of specific host leukocyte profiles and developmental features suggest that vaccination strategies, adjuvants, immunological responses, and approaches to ruminants and non-ruminants must be studied and elucidated within specific host species and *Leptospira* strain combinations.

## Scientific Challenges, Research Gaps and Potential Developments: *Leptospira* and Their Respective Reservoir Host

Research is needed to address the root persistence of leptospirosis and minimize disease transmission to incidental hosts. Emphasis should be placed on the study and characterization of the natural interactions between the pathogen and the reservoir host, both livestock and rodent alike, in the context of chronic disease. Though bacterin vaccines have inherent problems, efficacy can be improved. For instance, vaccine development should target not only specific serovars, but also specific *Leptospira* strains into vaccine formation which could utilize the unique relationships between host and *Leptospira* serovar and result in more host species-specific protection. Vaccine technology could also benefit from adopting a wider host model system. There is a need for understanding vaccine effects within the intended species host, not just the hamster model. To achieve this, phenotypes beyond virulence and survival must be established. This might include quantifying pathogen colonization and shedding, motility, adhesion, or other novel phenotypes that need to be developed in a non-terminal model such as the presence/absence and unique role of humoral versus cell mediated, and systemic versus local immune responses. Along with the intended species, vaccine models should focus on natural routes of exposure. Most hamster challenges involve intraperitoneal (IP) injection, which is not indicative of any natural route of infection. In fact, recent research has highlighted differences in pathogen kinetics, bacterial burden, and colonization between IP and more natural routes of experimental challenge, such as oral mucosa in mice (Nair et al., 2020). A hamster study also established that IP and conjunctival challenge methods, as well as organism dose, significantly impacted the dissemination and expansion of *Leptospira* (Wunder et al., 2016). Even intradermal and subcutaneous administration methods yielded survival and tissue burden differences in the hamster model (Coutinho et al., 2014). Conjunctival instillation is already routinely used for cattle experiments (Bolin et al., 1989b, 1991; Bolin and Alt, 2001) and similar approaches of natural routes of infection should be adapted to vaccine trials to best model and protect against the real-world environment of leptospirosis exposure. While domestic and livestock species rely on injection-based vaccines, bait-based vaccines must be adopted for vaccination of wildlife reservoir hosts of disease to truly control transmission of leptospirosis. Bait-based oral vaccines are an attractive option for targeting wild reservoir hosts of disease and greatly contributed to the elimination of rabies from foxes in Europe in the field (Müller et al., 2015). Bait-based vaccines have additionally proved effective for both experimental protection of mice from Lyme disease (Bhattacharya et al., 2011) as well as reducing the incidence of tick borne disease in field studies after vaccine deployment (Richer et al., 2014).

In addition to characterizing host immune-mediated pathways that tolerate persistent renal colonization by pathogenic leptospires, there is a need to understand what pathogenic mechanisms are employed by leptospires to subvert host-immune responses as well as maintain continued renal colonization and dissemination. The ability of leptospires to modify gene and protein expression in response to environmental cues during *in vitro* propagation, including those that mimic natural host infection, have been well established (Nally et al., 2001a,b; Lo et al., 2006; Matsunaga et al., 2007). Further, *Leptospira* cultured within dialysis membrane chambers (DMC), *in vivo*, in the intraperitoneal space of rats identified differentially expressed genes and highlighted physiological aspects of host adaptation by leptospires relating to heme uptake and utilization, as well as novel non-coding candidate small regulatory RNA expression (Caimano et al., 2014). However, as this review has established, gene expression and protein profiles of pathogenic leptospires *in vivo* are also predicated to be dependent on the infected host species, as well as the disease state (i.e., during an acute symptomatic versus persistent asymptomatic infection) (Nally et al., 2005). For instance, the same *L. interrogans* serovar Copenhageni responsible for severe pulmonary hemorrhage in the guinea pig caused an asymptomatic persistent kidney infection in the rat. Interestingly, the leptospiral LPS O-antigen, which has a role in adhesion and complement resistance, was poorly expressed by leptospires disseminated in acutely infected guinea pig tissues compared to expression by leptospires colonizing the renal tubules of experimentally infected rats (Nally et al., 2005).

Animal models of persistent renal colonization are readily amenable to the collection of urinary derived leptospires that are relatively free from host-derived protein compared to those disseminated in liver or kidney tissue (Nally et al., 2007; Monahan et al., 2008; Bonilla-Santiago and Nally, 2011). Urinary derived leptospires express a different proteome compared to their *in vitro* or DMC cultivated counterparts based on proteins expressed, but also differ by individual protein post-translational modification (PTM), including trimethyllysine and acetyllysine (Nally et al., 2011, 2017). Comprehensive bio-systems that characterize protein modifications by pathogenic leptospires have not only confirmed multiple PTMs, but also suggest that *L*. *interrogans* shares significant similarities with protein modification systems in eukaryotes (Cao et al., 2010). A comprehensive analysis of the transcriptome and proteome of urinary derived leptospires is required to understand pathogenic mechanisms of renal colonization and disease transmission. Furthermore, this would allow the identification of conserved protein antigens and their respective PTMs. These could then be additionally evaluated as determinants which interact directly with host immune-mediated pathways or could serve as potential cross-protective subunit vaccinogens.

Persistent disease transmission of leptospires is typically associated with colonization of renal tubules. However, colonization and adhesion within the genital tract, is also routinely observed in domestic animals including cattle, and as observed commonly with serogroup Sejroe (Ellis et al., 1986; Loureiro and Lilenbaum, 2020). Bovine leptospirosis can be used to study genital versus renal leptospirosis, though experimental work with cattle is largely under-represented in the literature, likely due to their size and housing needs, long gestational and growth timelines, and significantly greater cost.

Modeling the behavior of pathogenic leptospires in the laboratory can be inherently difficult given that *Leptospira* are notoriously sensitive to culture and *in vitro*-passaged leptospires can become attenuated with time. The use of *in vitro* cell culture assays to assess host–pathogen interactions requires consideration of the appropriate serovar and relevant host-derived cell line as implied throughout this review, as well as the use of low passage, virulent isolates. Most current culture systems fail to best portray true *in vivo* environments over sustained periods of time. For example, contrary to the relatively long generation times (6–16 h) for pathogenic leptospires *in vitro*, the generation time during renal colonization *in vivo* can be assumed to be much shorter given that naturally infected rats excrete on average 6.1 × 10^6^ leptospires per ml of urine (Costa et al., 2015b). Similar numbers can be observed in experimentally infected rats over several weeks which can excrete more than 15 ml of urine overnight (Monahan et al., 2008). A better understanding of metabolic requirements for leptospires during renal colonization would not only enhance our ability to culture leptospires *in vitro*, but also provide insight into the development of culture systems that are more closely aligned with the *in vivo* infection environment. For instance, recent developments in the field of media composition have allowed, for the first time, successful long-term culture of *L. borgpetersenii* at 37°C, which better represents the incubated environment of live host (Hornsby et al., 2020).

Utilizing the natural reservoir host provides an opportunity to directly address some of the largest gaps in current leptospirosis research, and also contribute to models of acute disease. Such models also provide the opportunity to address clinical cases whereby acute symptoms resolve to asymptomatic carriage. This is evident not only in animals, but also in humans. Case reports demonstrate the ability of dogs to persistently shed leptospires even after antibiotic treatment (Juvet et al., 2011; Mauro and Harkin, 2019). Unique human cases have also been described, where asymptomatic shedding can occur for extended periods of time (Ganoza et al., 2010; Chow et al., 2012).

Studying reservoir host systems addresses the naturally occurring issues of persistence and transmission of disease. Currently available tools should be utilized to study in detail relevant and naturally occurring reservoir host/serovar relationships. The widespread nature of *Leptospira* and the wide range of zoonotic hosts makes complete disease eradication unrealistic. However, the use of reservoir hosts as animal models of persistent infection provide the potential for leptospirosis research to advance our understanding of disease pathogenesis, transmission dynamics, immune responses, immune escape by the pathogen, and *Leptospira* and host species interactions which are critical to understand in the context of zoonotic control.

## Author Contributions

EP wrote the manuscript. JN contributed greatly to the content of the review and edited the manuscript. Both authors contributed to the article and approved the submitted version.

## Conflict of Interest

The authors declare that the research was conducted in the absence of any commercial or financial relationships that could be construed as a potential conflict of interest.

## Footnotes

1 https://www.ncbi.nlm.nih.gov/pubmed
